# Land Efficient Mobility: Evaluation of Autonomous Last Mile Delivery Concepts in London

**DOI:** 10.3390/ijerph191610290

**Published:** 2022-08-18

**Authors:** Maren Schnieder, Chris Hinde, Andrew West

**Affiliations:** The Wolfson School of Mechanical, Electrical and Manufacturing Engineering, Loughborough University, Loughborough LE11 3TU, UK

**Keywords:** time-area concept, space efficiency, land efficiency, last mile delivery, land consumption, autonomous delivery, parcel lockers, CDPs

## Abstract

Land efficient last mile delivery concepts are key to reducing the traffic in cities and to minimising its environmental impact. This paper proposes a decision support method that evaluates the autonomous delivery concept and applies it to one year’s worth of real parcel delivery data in London. Deliveries to modular and fixed lockers with autonomous delivery vans and road-based autonomous lockers (RAL) and sidewalk autonomous delivery robots (SADRs) have been simulated. Various types of autonomous delivery van fleets, depot locations, customer modes of transport, parcel demand levels, parcel locker network densities and adjustment frequencies of modular lockers are considered. A routing and scheduling algorithm is used to optimise delivery tours and vehicle choice. The optimisation algorithm finds both the optimal number of collection and delivery points (CDPs) and the delivery concept (e.g., modular lockers, sidewalk autonomous delivery robot) depending on the customer mode chosen. The results show that modular lockers which are adjusted weekly are the best option for the current or higher parcel demand levels and road-autonomous parcel lockers (RAL-R) are the best option at the lowest parcel demand level.

## 1. Introduction

Due to the growth in both the urban population and e-commerce [1], an increasing amount of road space is devoted to last mile delivery vehicles [2]. These delivery vans slow down traffic in cities, which causes congestion [2], reduces the space available in cities [3,4] and increases emissions [5,6]. Hence, there is a need for alternative last mile delivery options [7] to increase the sustainability of last mile deliveries. Autonomous vehicles and autonomous delivery robots (ADRs) have received much attention [8,9]. Delivery companies see ADRs as an option to reduce cost, delivery mistakes, delivery time and energy consumption [10], to improve safety for personnel [10], to increase delivery efficiency through 24/7 delivery [11], to increase eco-friendliness [11] and to reduce traffic fatalities [11]. On-demand meal delivery companies in particular have shown an interest in deploying ADRs in recent years [10].

The interest in ADRs is not new. For example, in 2014, DHL proposed a self-driving vehicle that follows the courier to reduce the number of times the courier has to go back and forth between the van and the customers [12]. A lack of availability of parking spots close to the customer is a major cause of inefficiencies in the delivery process due to the walk to the customers’ premises, which can be an issue for personnel, especially when the parcels are heavy or bulky [12]. DHL also proposed the concept of ‘self-driving parcels’, which are now known as sidewalk autonomous delivery robots (SADRs). Another future solution suggested by DHL was a ‘self-driving Packstation’. This concept represents a mobile parcel locker that stops at designated locations for a specific duration to allow customers to collect their parcel(s) [12].

While the land consumption of autonomous vehicles for passenger transportation is a major concern, no study has compared the land consumption of various types of ADRs combined with modular or fixed lockers. However, reducing the space requirements of transport activities and using space effectively in urban areas is key to alleviating increased congestion, parking pressure, the area required for streets and allowing more space for housing in urban areas [13].

The contribution of this paper is a decision support method to evaluate the time-area consumption levels (i.e., space efficiency) of various autonomous last mile delivery services. The decision support method is applied to a real-world dataset comprising one year of parcel delivery trips made by a delivery company in London, UK, during which two-million parcels were delivered. This dataset allows for a more realistic simulation, as opposed to a probabilistic simulation, given that every outlier has been simulated as well. Every day of the year has been simulated assuming that all of the parcels are either delivered by road-based autonomous lockers (RAL), SADRs or autonomous delivery vans (ADV) which deliver parcels to fixed or modular lockers, and are adjusted quarterly, monthly, weekly or daily. Two different depot locations are considered (i.e., rural depot and city depot). All trips, including the parcel delivery trips, modular locker adjustment trips and customer trips, are considered. Ten different demand levels and twelve different parcel network densities have been simulated. An optimisation algorithm has been used to find the optimal delivery concept (e.g., numbers of collection and delivery points (CDPs) and delivery vehicles) depending on the customer mode choice. The decision support method proposed in this paper is especially useful for policymakers to gain an understanding of the land consumption of various autonomous delivery services in order to allocate research funding to the most suitable option, and later, once various ADRs are developed, they can find the best method to implement these. Minimising space consumption in cities is key to alleviating issues caused by the limited space in cities, such as traffic jams, a housing shortage and a lack of parking spaces and bike or bus lanes.

## 2. Literature Review

### 2.1. Autonomous Delivery

Autonomous vehicles for passenger transportation have been extensively researched, but research focused on autonomous vehicles used for freight transport is limited [9,14,15]. A large number of studies are focused on technical aspects, routing or scheduling policies [10]. Examples include Silvestri et al. [16], who simulated an ADR to analyse the dynamic performance; Boysen et al. [17], who focused on developing a scheduling process for truck-based ADRs; Bärtschi et al. [18], who proposed a scheduling algorithm for energy constrained ADRs; and Ulmer et al. [19], who modelled same-day delivery to parcel lockers by ADRs as a Markov decision process and proposed a real-time dispatching decision function.

Some studies evaluated the performances of ADRs such as Figliozzi [9] and Vleeshouwer et al. [20]. Figliozzi [9] analysed the energy consumption and CO_2_ emissions of unmanned aerial vehicles (UAVs), SADRs and road autonomous delivery robots (RADR) compared with an electric van (EV) for use in last mile and grocery deliveries. Based on their calculations, non-truck-based SADRs are the best option for small operating areas; UAVs perform best in low density and time-constrained deliveries; and RADRs are a better option than EVs if the number of customers is relatively low. In a similar study, Figliozzi et al. [10] concluded that RADRs perform better than SADRs from an environmental view point. Figliozzi et al. [15] concluded that RADRs can increase congestion due to their larger travel distance per customer served. SADRs have relatively long delivery times, given that they can only deliver one package at a time, and therefore, return to the depot after every delivery trip. For small fleet sizes (i.e., less than 4), RADRs were less expensive than conventional vans (i.e., 0.1$/customer reduction); conventional vans were less expensive (i.e., 0.1$/customer reduction) when the delivery tours were large and the density high, and the tour could be fulfilled with one van. SADRs were never the cheapest option in any of the scenarios. While Figliozzi [9], Figliozzi et al. [15] and Figliozzi et al. [10] calculated the results, they did not simulate scenarios based on real city requirements and infrastructures. Vleeshouwer et al. [20] simulated a bakery delivery service and concluded that SADRs can reduce the cost by 40% due to the reduced staff cost compared with a real-world case study using a delivery van. However, replacing the delivery van with SADRs in the case study was not profitable, given that they delivered only one day a week, resulting in a low utilisation.

Acceptance of ADRs has been studied by Kapser et al. [7], who concluded, based on a survey of 501 participants, that price sensitivity is the most important factor for user acceptance, followed by performance expectancy, risk, social influence and hedonic motivation. The regulations, abilities and benefits of SADRs have been studied by Jennings et al. [21], such as reductions in on-road travel, which alleviate congestion. However, by traveling on footpaths, the SADRs are a safety concern for pedestrians and could cause congestion on footpaths.

### 2.2. Land Use Efficiency

**Figure 1:**
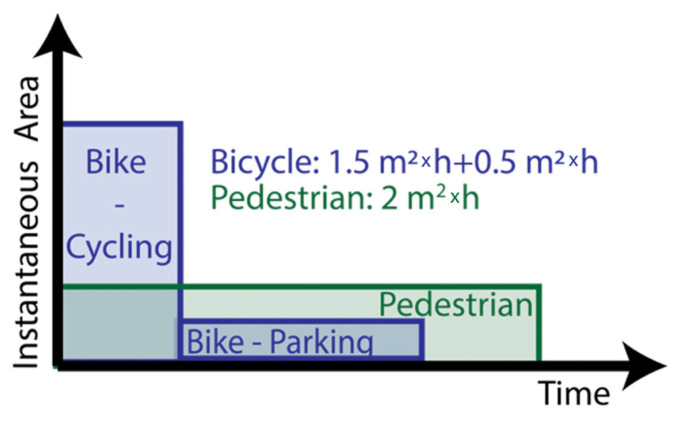
Time-area requirements of bicycle (blue) and pedestrian (green) (source: [13]).

The decision support method used in this paper is based on the time-area concept, which combines the space requirements of ground-based transport units while moving and parking into one metric [13] and is defined as the ‘ground area consumed for movement and storage of vehicles, as well as the amount of time for which the area is consumed’ [22] In other words, the time-area requirement measures the area required for a transport activity and the duration for which it is occupied. Figure 1 illustrates the calculation of the time-area requirements. A pedestrian might travel for 1 h and requires 2 m^2^ to move comfortably during this time. While a cyclist might travel four times as quickly as a pedestrian and can cover the same distance in 15 min, the cyclist requires a larger area to move comfortably, e.g., 6 m^2^. Hence, the time-area requirements of the pedestrian and cyclists are 2 m^2^ × h and 1.5 m^2^ × h, respectively. In contrast to a pedestrian, a cyclist must store the bicycle after a trip, which increases the time-area requirement by, for example, an additional 0.5 m^2^ × h for a 30 min stopover [13].

The time-area concept combines the land consumption of moving and parking vehicles into one, making it a perfect choice for autonomous vehicles, which can replace parking requirements by continuing to drive [4]. The metric can be used to compare various modes of transport which have different sizes or velocities [13]. The time-area concept also disregards the financial value of land, as otherwise all traffic would be diverted into poor neighbourhoods, even if it is a detour due to the lower land value [13].

For an extensive review of the time-area concept, the reader is referred to Schnieder et al. [13]. One of the first detailed evaluations of the time-area concept was probably the Ph.D. thesis by Bruun [22]. Later, Brunner et al. [23] compared the instantaneous area requirements of transport activities per person. While they did not mention the term ‘time-area concept’ in their paper, they provided a detailed explanation of their methodology. Shin et al. [24] proposed an equation to calculate the time-area requirements using the continuous flow formulation. Additionally, Bruun et al. [25] published a continuous flow formulation of the time-area concept a few years earlier. The two proposed equations can be transformed into each other using the fundamental stream equation [26]. Other researchers only published tables with time-area requirements without a detailed explanation of the assumptions or equations used (e.g., Litman [27]). The time-area concept is mentioned in anecdotal books, such as ‘Happy City: Transforming our Lives Through Urban Design’ [28] or ‘Sustainable Parking Management’ [29]. However, these books only provide examples of the time-area requirements with many assumed details which were not specified. This reduces their value for policy decisions [13]. Given that the scientific publications generally focus on the mobility of people and not on last mile delivery [13], the authors published a series of papers to close this gap. Schnieder et al. [13], reviewed the published literature about ways to measure the space efficiency of transport activities and developed a method to quantify it using the time-area concept. This decision support method has been applied to a one-year long dataset of last mile delivery trips in London. Schnieder et. al. [4] compared the time-area requirements of on-demand meal delivery using delivery robots, bicycle couriers or delivery vans. Various operating strategies (e.g., shared fleets), parking policies and scheduling options (e.g., tour-based delivery and direct delivery) have been simulated in New York City (NYC). They also used GPS traces of Deliveroo and UberEats riders to estimate the time-area requirements. The authors concluded, for example, that sidewalk autonomous delivery robots (SADRs) can reduce the time-area requirements by half compared with bicycle couriers.

## 3. Methods

### 3.1. Dataset, Demand Estimation and Network

#### 3.1.1. Real Demand Data Set

A dataset of 13,358 delivery trips by Gnewt in London [30] during which 2 million parcels were delivered between July 2015 and June 2016 has been used to determine the number of parcels per day. The number of collected parcels had to be set to 0 for 8 of the delivery trips due to errors in the data entry. Based on this dataset, a list of successfully delivered and picked up parcels (collections and pickups) for each day of the year has been created. Unsuccessful delivery or attempted pickups were disregarded, as it was assumed that these would be delivered the next day; otherwise, the parcel would be counted twice. The demand dataset (i.e., number of parcels for each day of the year) is calculated as follows:

(1) $$n_{p_{d}}=n_{d_{d}}+n_{r_{d+1}}+n_{f}{}_{d-1}$$

where,

$n_{p_{d}}$ Number of parcels in the system on day d;

$n_{d_{d}}$ Number of successful parcel deliveries on day d;

$n_{p_{d+1}}$ Number of pickups by a delivery driver on the following day (return deliveries);

$n_{f_{d-1}}$ Number of parcels not picked up by customers on the previous day.

The number of pickups was added to the previous day’s number of parcels, as customers would have needed to schedule the pickup on the previous day. Note: in an autonomous and on-demand delivery system, it would have been possible to pick any parcel up immediately. Additionally, if a parcel is picked up from a locker by a driver, it must have been placed in the locker during the previous 24 h. Thus, the returned parcel number needs to be added to the number of required lockers on the previous day. The number of parcels that have not been picked up within 24 h after the delivery is assumed to be 30%, as suggested by a German delivery company [31]. To accommodate longer delays when parcels are left within lockers, it is assumed that 9%, 2.7% and 0.8% of parcels are left in the locker for 2, 3 and 4 days, respectively, as in reference [32]. The parcels which are not picked up are chosen randomly.

Note: It is possible that the demand for parcels will increase or decrease in the future. Hence, the simulations were also run assuming that only 20% of the currently delivered parcels were delivered. This might represent the first year when the parcel delivery service was implemented. An 80% increase in the parcel demand was also simulated, as the demand could significantly increase in the future. The simulation has also been run assuming that 20%, 40%, 60%, 80%, 100%, 120%, 140%, 160%, 180% or 200% of the parcels have been delivered. The results of this are reported in 4.3.

#### 3.1.2. CDP Network

The resulting demand dataset (i.e., number of parcels per day for one year) was used as the input for the simulations in this study. GNEWT delivers parcels to customer homes/work addresses within London. The exact delivery locations are unknown to the authors.

In this simulation, it was assumed that the parcels were delivered to CDPs. Twelve different CDP networks have been created. Each of the 12 networks has a different number of CDPs, namely, 1, 2, 4, 8, 16, 32, 64, 128, 256, 512, 1024 and 2048. In each of the 12 CDP networks, the CDPs were randomly placed around the operating area with the largest possible minimum distance between two CDPs for which the QGIS [33] function Random Points inside Polygons (fixed) was able to find a solution. This approach was chosen to ensure that the CDPs were spread out as evenly as possible when using random allocation. Placing CDPs randomly is a commonly used method (see e.g., [34]). Note: Some CDPs were place in inaccessible places, such as the river. The routing algorithm automatically assumes that each CDP is accessed from the nearest point on a street.

**Figure 2:**
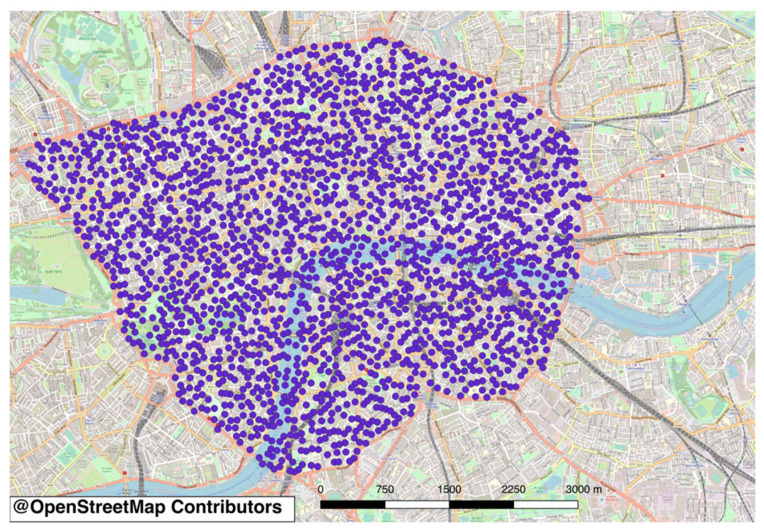
Map of the 2048 CDPs in the study area.

The operating area in the simulation (i.e., CDP network coverage area) is the congestion charge zone in London (Figure 2), which is the main delivery area of GNEWT [35].

The number of parcels per day is then randomly allocated to the CDPs. In detail, $n_{d_{d}}$ (i.e., number of successful parcel deliveries on day d) and $n_{p_{d+1}}$ (number of pickups by a delivery driver on the following day (return deliveries)) were randomly allocated to the CDPs. $n_{f_{d-1}}$ (i.e., number of parcels not picked up by customers on the previous day) was determined by adding 30% of the parcels that are in each of the CDPs on the previous day, to the number of parcels for the following day in the same CDP. The same method has been used in Schnieder et al. [32].

#### 3.1.3. Size of Each CDP within the CDP Network

**Figure 3:**
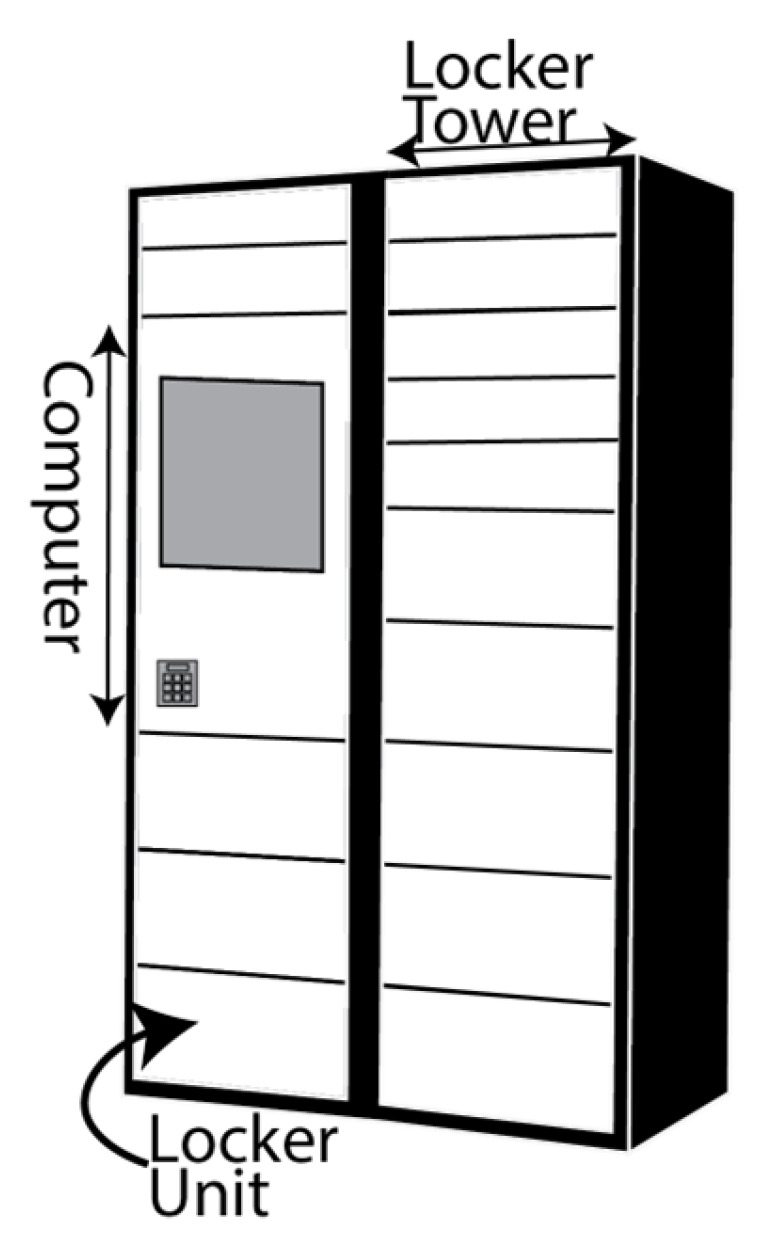
Parcel locker.

The fixed and modular parcel lockers were assumed to have a similar size to typical Amazon lockers (see Figure 3). A parcel locker unit which can fit one parcel occupies an area on the ground of 45 × 45 cm^2^, and 10 parcel locker units (i.e., compartments) can be placed above each other. Note: In reality, the height of these individual parcel locker units varies to accommodate different parcel sizes, as shown in Figure 3. However, to simplify the simulation, it is assumed that each parcel fits in the allocated parcel locker unit (i.e., the parcel size has been ignored). The computer to operate the parcel locker has the size of five locker units. One computer is installed at every CDP, regardless of how many lockers units are installed. Given that in the future, when the proposed automated delivery concepts might be operational, locker units could open automatically when the customer approaches the locker with his or her phone. This is similar to cars unlocking themselves when the driver goes near the car with his or her key.

The required number of parcel locker spaces is calculated based on:

(2) $$n_{t_{\mathrm{CDP}}}=\mathrm{ceil}\left(\frac{\left(\underset{1\leq d\leq D}{\max(}n_{p_{{\mathrm{CDP}}_{d}}})+s_{c}\right)}{s_{h}}\right)$$

where,

$n_{t_{\mathrm{CDP}}}$ Number of locker towers at a specific CDP;

$n_{p_{{\mathrm{CDP}}_{d}}}$ Number of parcels at specific CDP on day d;

d Current day of time interval;

D Last day of the time interval;

$s_{c}$ Size of computer in locker units (i.e., 5);

$s_{h}$ Number of locker units above each other (i.e., 10).

### 3.2. Delivery Concepts

The sizes of vehicles used in this study and numbers of parcels that can fit in them were estimated based on average size and weight values. An SADR can usually fit 1 parcel [11]. Small vans (sv), such as the Renault Kangoo, Fiat Doblo, Ford Transit Connect and Volkswagen Caddy, can usually transport up to 770 kg of weight and 3.3 m^3^ of volume, based on the websites of the manufacturers. Medium vans (mv) are short wheelbase (SWB) vans such as the Ford Transit Custom, Renault Traffic, Mercedes Vito and VW Transporter. These can carry up to 1 t and a volume of 5.6 m^3^, based on the websites of the manufacturers. Next, 3.5 t vans such as the Ford Transit L3, Renault Master, Mercedes sprinter L2 and VW Crafter can usually transport up to 1.6 t and 9.6 m^3^, based on the websites of the manufacturers. Next, 7.5 t trucks such as the Fuso Canter (Daimler group), Mercedes Atego, Isuzu forward, and IVECO ML can load up to 3.8 t and 34 m^3^ usually, based on the websites of the manufacturers. These are similar the load capacities used in other studies, such as [36,37,38].

**Figure 4:**
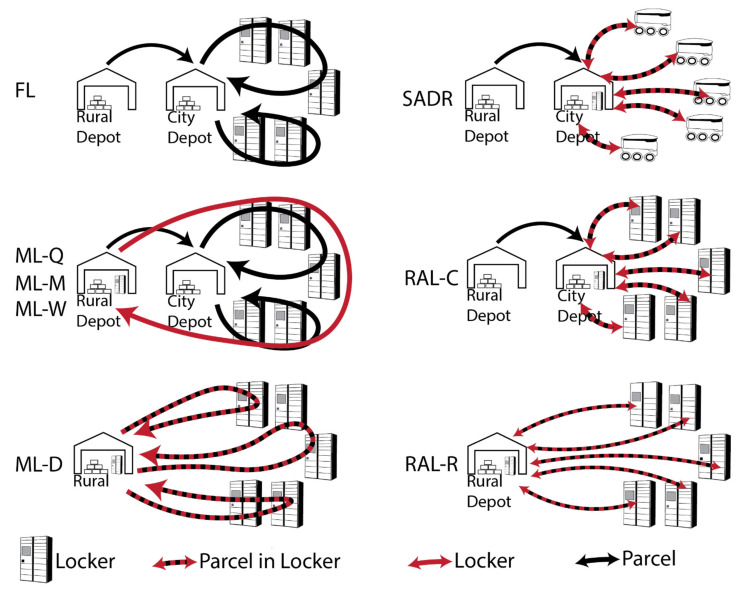
Delivery concepts: Fixed locker (FL), modular locker adjusted quarterly (ML-Q), modular locker adjusted monthly (ML-M), modular locker adjusted weekly (ML-W), modular locker adjusted daily (ML-D), sidewalk autonomous delivery robot (SADR), road-based autonomous locker parked at city depot (RAL-C), road-based autonomous locker parked at rural depot (RAL-R).

Figure 4 illustrates the vehicle and locker configurations considered in the study.

FL comprising fixed lockers + van: Parcels are delivered and picked up by a driverless van. The vans depart from and return to a city depot. The number of required locker units is fixed and equal to the maximum number of locker units required at each CDP over the year. During traditional home delivery, the driver spends, on average, 62% of the total round-trip time walking between the van and the customer’s home [39]. However, for the case of autonomous delivery to parcel lockers, it is assumed that the delivery vehicle is able to stop directly in front of the parcel locker, and it takes five seconds to place one parcel into a locker. Note: this could also be achieved by having five robotic arms in parallel needing 25 s per parcel. It is also assumed that it takes five seconds to load each parcel into the van. Parcels that are not retrieved by the customer are left in the locker for the next day.

ML-Q comprising modular lockers adjusted quarterly + van: Parcels are delivered and picked up by an autonomous delivery vehicle, which is based at the city depot. In this scenario it is assumed that it takes 5 s to place a parcel into a locker or to load it into the van. The modular parcel locker capacity is adjusted every 3 months, and the number of parcel locker towers (i.e., totalling 10 locker units) is equal to the maximum number of required locker towers at each CDP during each time period. As only 1 year of data were available for the basis of this analysis, the modular parcel locker capacity that was already in the city at the start of the simulation was assumed to be the same as at the end of the simulation (4th quarter). It was assumed that, based on the previously mentioned load capacity of a 7.5 t truck and the average locker size, a 7.5 t truck can transport 65 locker towers. The number of trucks required for each locker adjustment was based on the difference between added locker towers and removed locker towers. Each truck visits all CDPs to pick up and drop off locker towers as required. Locker delivery trucks travel to and from the city depot. Parcels that have not been collected by customers are left in the locker. Note: The time-area contribution of the locker modification trip is an approximation: It is assumed that each truck travels in an optimal route past each CDP and takes 20 s to modify the locker, similarly to the time it requires nowadays for automated dustcart lorries (i.e., garbage truck) to empty a wheelie bin (e.g., 4–5 s [40,41]) or garbage container (e.g., 80 s [42]).

ML-M comprising modular lockers adjusted monthly + van: The same as ML-Q, but the lockers are adjusted monthly.

ML-W comprising modular lockers adjusted weekly + van: The same as ML-Q, but the lockers are adjusted weekly.

ML-D comprising modular lockers adjusted daily: Each day, the required number of locker units, which are already filled with parcels at the rural depot, are dropped off at each CDP. The locker units are collected again after a 14 h time window (which is similar to supermarket opening times), during which customers can pick up their parcels. Uncollected parcels are redelivered the next day. Locker units are delivered instead of locker towers to avoid the delivery and return of half-empty locker towers. The required area of the standing ML-D is determined based on the assumptions that the locker units are stacked as locker towers. The computer is assumed to be left at the CDP overnight, and the time-area requirement is calculated over a period of 24/7. It is assumed that 650, 240, 120 and 73 locker units can be transported in 7.5 t, 3.5 t, medium and small vans, respectively, based on the previously mentioned load capacities.

SADR-0: Sidewalk autonomous delivery robots (SADRs) travel at walking speed from the city depot to a CDP to deliver or pick up one parcel at a time. It is assumed that this service takes place for 14 h per day and that the SADRs have no waiting time between deliveries. It is also assumed that the SADRs wait an average of 5 min at the CDP, which includes the time the customer takes to arrive at the CDP and to take the parcel out of the SADR. Given that this is an on-demand service, it is assumed that all customers pick up their parcels, in contrast with RALs, ML and FL where the model assumes that 30% of the customers do not pick up their parcel on the delivery day.

SADR-15: This simulation is the same as SADR-0, but the SADR has to wait on average 15 min between parcel deliveries due to fluctuations in customer demand and delays.

SADR-45: The same as SADR-0, but the SADRs have to wait on average 45 min between parcel deliveries due to fluctuations in customer demand and delays.

RAL-C: Road-based autonomous lockers (RAL) drive each day from the city depot to each CDP and park there for 14 h until they return to the city depot. The number of RALs available in the system is equal to the maximum number of RALs required on any given day. Unused RALs remain at the city depot. Uncollected parcels are redelivered the next day. Given that RALs would be too large to travel on footpaths, it is assumed that they will travel on roads with the same average speed as cars to ensure that they will not disrupt the traffic flow.

RAL-R: This simulation is the same as RAL-C, but RALs start from and return to the rural depot each day.

Depot trips: All parcels are transported by one or multiple vehicles from the rural depot to the city depot in all simulations apart from ML-D and RAL-R. It is assumed that it takes 1 min to load and unload one batch of 300 parcels on a palette into the delivery truck. This is in line with current and future developments of automated loading/unloading of trucks [43,44,45]. The loading procedure is automated and both the rural depot (i.e., departure point) and city depot (i.e., arrival point) have specialised loading and unloading facilities.

### 3.3. Delivery Trip Routing and Customer Trips

The model parameters for customer trips were estimated based on walking, cycling and driving distances between all active postcodes (17,691) [46] within the chosen operating area in London and their nearest CDP. The trip distance and duration for every mode of transport were calculated based on a locally hosted open-source routing machine (OSRM) [47] and Open Street Map (OSM) street network data [48].

The delivery tour routing has been performed using a locally hosted open-source routing machine (OSRM) [48] and Open Street Map (OSM) street network data [48]. The simulation has been implemented in Python, and the figures have been created using the Python libraries seaborn [49] and matplotlib [50]. Other common Python libraries, including random, pandas, numpy, requests, json, ast, itertools and glob, have been used. In the simulations presented in this paper, four vehicles (7.5 t truck, 3.5 t truck, medium van (mv), small van (sv)) were available to deliver the parcels to fixed lockers (FLs) and modular lockers (MLs). The developed tour scheduling and routing algorithm clusters the CDP deliveries into trips based on their geographic locations to ensure that close by CDPs are delivered by the same delivery vehicle. Each vehicle has a maximum number of parcels it can carry and can visit a maximum of 300 CDPs daily, as otherwise, the delivery duration would be too long. If the number of parcels to be delivered to the same CDP is larger than half the capacity of the van, the algorithm will send smaller delivery vans directly to the relevant CDPs. This constraint has been added since it is more effective in terms of time-area savings to send two small vans to two CDPs compared with sending a large truck first to one CDP and then to the next when the CDPs are far apart. Even if the instantaneous area requirement of using two small vans is larger compared with using one large van, using one large van can take twice as long to complete the deliveries if the CDPs are far apart. Note: When the number of parcels per CDPs is very high (e.g., half a truck load), then only a few CDPs exist in the system, meaning the two CDPs will be rather far apart. Once the algorithm has created the delivery schedule for the day, for each tour the smallest available vehicle that can fulfil the tour is selected.

The city depot has the coordinates −0.125376, 51.516637, and the rural depot has the coordinates 0.042561, 51.575093. The distance between them is around 16.2 km. All parcels were transported by trucks from the rural depot to the city depot each day in all simulations, apart from ML-D and RAL-R. The number of required delivery vehicles has been calculated based on the following equation:

(3) $$n_{{7.5t}_{d}}=n_{p_{d}}/c_{7.5t}$$

(4) $$R=n_{p_{d}}\;mod\;c_{7.5t}$$

where,

$n_{{7.5t}_{d}}$ Number of 7.5 t trucks on day d;

$n_{p_{d}}$ Number of parcels on day d;

$c_{7.5t}$ Number of parcels that can be loaded into a 7.5 t truck;

R Remaining of parcels that are delivered in the smallest van they fit in.

The van used to transport R—the remaining parcels—is the smallest van with an appropriate capacity. Even using two small vans requires a larger time-area than using one 7.5 t van (Table 1), given that all trucks travel to the same delivery destination.

### 3.4. Time-Area Requirements

It is important to use any land efficiently, regardless of whether it is in an expensive or poor neighbourhood, in a rural area or in the city centre [13]. However, in reality, policymakers and companies pay more attention to the city centre, given that more people live or work in the city centre, and therefore, more people are affected by it. Additionally, CBD are usually crowded, land is expensive and every extra square meter devoted to last mile delivery is lost for housing or parks. Thus, in this research only the time-area requirements within the CBD are considered, and not the time-area requirements at the rural depot.

**Figure 5:**
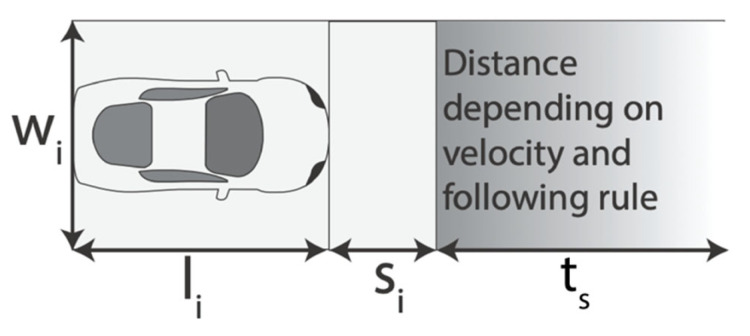
Specifications of the simulation (source: adapted from [13]).

The time-area requirements are calculated based on Equation (5) or (6). The equation can be used for congested flow, free-flow and parking. The equation only considers the legally required area for the transport activity and not the area that is actually occupied. The equation includes the 2 s separation distance as required in most parts of Europe and a safety distance $s_{i}$. A parked car would have no gap in front of the front vehicle when parking without the safety distance $s_{i}$, given that the 2 s separation rule only works for moving vehicles. For a detailed explanation, the reader is referred to [13]. Figure 5 illustrates the components of the equations:

(5) $$TA_{i}=\left(l_{i}+s_{i}+\left(\frac{t_{s}\times d_{i}}{t_{i}}\right)\right)\times w_{i}\times t_{i}$$

which can be reduced into:

(6) $$TA_{i}=\left(\left(l_{i}+s_{i}\right)\times t_{i}+\left(t_{s}\times d_{i}\right)\right)\times w_{i}$$

where,

$TA_{i}$ Time-area required for trip;

$l_{i}$ Length of vehicle;

$s_{i}$ Safety distance between standing vehicles;

$d_{i}$ Trip distance;

$t_{i}$ Trip duration;

$w_{i}$ Width of the lane/right-of-way;

$t_{s}$ Headway/following rule (usually two seconds).

Table 1 shows the specifications for the time-area simulation adapted from [13]. The safety margins around the RAL were 0 mm, as it is assumed that several RALs can interlink and travel together in a convoy. For traditional vehicles (i.e., not autonomous vehicles), the width of the required instantaneous area is usually assumed to be the same as the width of the right of way. In a fully autonomous transport system, it would be possible for vehicles to travel in a convoy, and the width of the right of way could be adjusted depending on the requirements. Hence, only a limited safety margin is required to the side of the RAL.

The maximum numbers of locker units that can fit are 73, 120, 240, and 650 for small vans, medium vans, 3.5 t vans, and 7.5 t trucks using the previously mentioned typical carrying capacities of vans and the sizes of the locker units detailed earlier.

This study did not consider the parking of autonomous delivery vans at the depot, as it was assumed that these vehicles can be used 24/7 for deliveries and other purposes. If they were to be electric vehicles, they would be charged by swapping batteries and not by charging the batteries. Having an entire vehicle sitting around just to charge the battery would not be efficient from a time-area perspective. This assumption demonstrates the best case that autonomous delivery vans can achieve in which they are continuously in operation. If a van is not used for deliveries, it is assumed that it is used for alternative business. As already shown in the other studies, parking at the depot is one of the largest contributors to the time-area requirements [13]. However, this does not apply to RALs and SADRs, as they are most likely built for purpose and cannot be used for anything else and are only useful during daytime hours due to the required interactions with customers.

## 4. Results

### 4.1. Time-Area Requirements: Customer Trips

**Figure 6:**
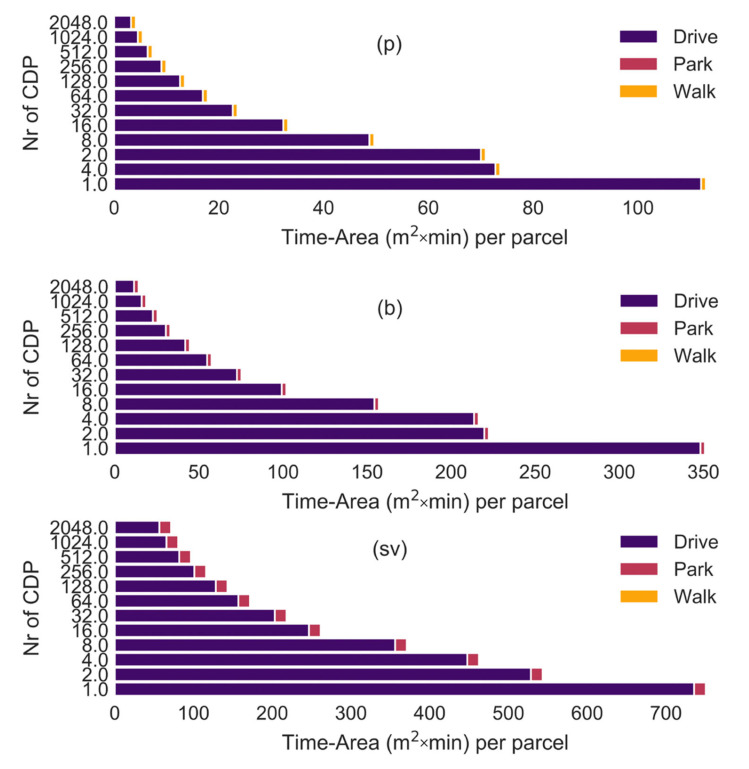
Time-area requirements of trips to lockers by customers (p) pedestrians, (b) bicycles, (sv) small vehicles. Number of CDPs varied from 1 to 2048.

The twelve CDP networks modelled in this study represent a range of parcel locker network densities collectively similar to the spatial distribution of parcel lockers in Germany [31]. In this study, the average roundtrip walking distance between the customer’s home and CDP ranges from 4.7 km (1 CDP) to 135 m (2048 CDPs) for pedestrians, 5.2 km (1 CDP) to 160 m (2048 CDPs) for cyclists and 6.0 km (1 CDP) to 435 m (2048 CDPs) for car drivers. The distance differs, as each mode of transport can use a different street network, and one-way streets can increase the short distances for drivers. This effect is reduced the further the customer has to travel to the CDP. Having 16 CDPs results in an average roundtrip distance between the CDP and the customer’s home of around 1.3 km. Note: the one-way trip distance is 600 m for residents in German cities (=1200 m round trip) and 3 km (=6 km round trip) in rural areas [31]. The time-area requirements for customers are illustrated in Figure 6. As expected, the time-area requirements increased with a reduction in the CDP density, and the time-area requirements of small vehicles (sv) were around seven times larger than for pedestrians. The majority of the time-area requirements are caused by the customer moving from their home to the CDPs and back. Pedestrians do not have any parking requirements. The time-area requirement for customers to walk from their parked bicycle or car to the CDP is so small that it is almost invisible in Figure 6(b) and Figure 6(sv). The distance to CDPs was larger for pedestrians when four CDPs were used compared to when two CDPs were used due to the locations of the CDPs, which was determined by the Random Points inside Polygons (fixed) in QGIS using the largest possible separation.

### 4.2. Time-Area Requirements: Delivery Vehicles

The time-area requirements for deliveries have been sub-divided into the following components:

DT-Un/Loading: Loading and unloading parcels into the vehicle which transports all parcels from the rural depot to the city depot.
DT-Driving: Driving all parcels from the rural depot to the city depot (roundtrip).
Depot-Standing: The time the SADRs and RALs are standing unused at the city depot.
Loading: Loading the parcel into the delivery vehicle.
Driving: Driving the delivery vehicle.
Parking: Placing the parcel into a locker.
R-Driving: Drive the ML-D back to the rural depot after they have been in the city centre for 14 h.
R-Parking: Load ML-D in the city centre onto delivery vehicles.
R-Unloading: Unload ML-D at the rural depot.
L-Standing: The time the lockers are standing either at the city depot or in the city centre.
L-Moving: Lockers transported from the rural depot to their respective CDP.
L-Parking: Unloading/loading the lockers at the CDP.

**Figure 7:**
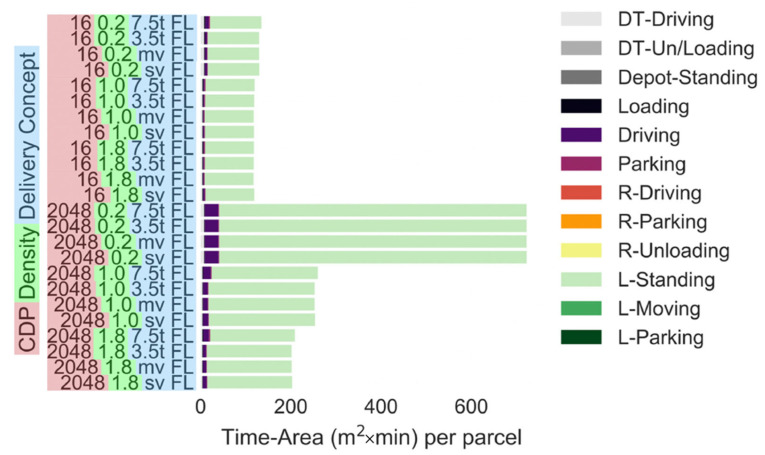
Time-area requirements of delivery to fixed lockers assuming various maximum delivery vehicle sizes (number of CDPs: 16 or 2048). Parcel demand level: 0.2: 20% of the current demand, 1.0: current demand, 1.8: 80% increase in the demand. (Note: All parts apart from driving and L-Standing are negligibly small).

Figure 7 compares delivery to fixed lockers with various maximum delivery van sizes (i.e., 7.5 t truck, 3.5 t truck, medium van and small van). The simulation was conducted with CDP networks of 16 and 2048 CDPs. The fixed parcel lockers account for the largest share of the time-area requirement (i.e., 16 CDPs: 109 m^2^ × min, 2048 CDP: 235 m^2^ × min). The utilisation of the lockers is as low as 30–40% due to the seasonal changes in the parcel demand. The utilisation reduces with an increase in the number of CDPs [32]. Excluding larger delivery trucks (i.e., 7.5 t) from the pool of possible delivery vehicles to choose from only reduces the time-area requirements of the delivery vehicle driving and parking by around 33%. Using only small vans to deliver parcels increases the time-area requirements by 10%, given that more vehicles are required. However, these reductions or increases are negligible in comparison to the large time-area requirements of the fixed locker.

**Figure 8:**
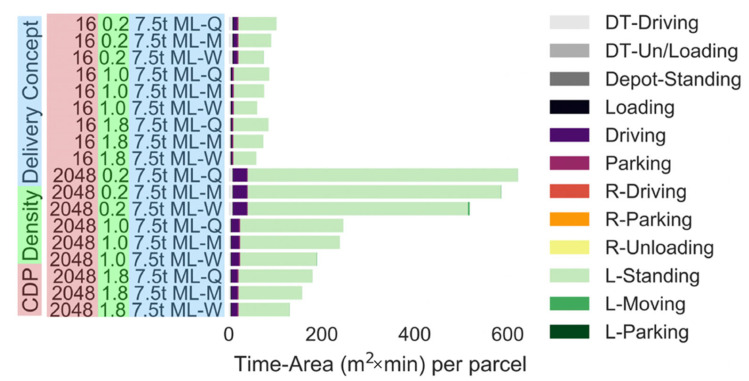
Time-area requirements of modular lockers (number of CDPs: 16 or 2048). Parcel demand level: 0.2: 20% of the current demand, 1.0: current demand, 1.8: 80% increase in the demand. (Note: All parts apart from driving and L-Standing are negligibly small).

Modular lockers can reduce the time-area requirement, since the number of lockers can be adjusted based on the demand. The time-area requirements of lockers can be reduced by adjusting the locker quarterly (1–31% reduction), monthly (9–40% reduction) or weekly (30–55% reduction); see (Figure 8). All locker capacity modifications in the simulations can be performed with one or two 7.5 t trucks. Thus, the time-area requirement of the locker adjustment trip (i.e., as low as 1 m^2^ × min per parcel) can be ignored when viewed on a per parcel basis. Note: the simulation assumed that unused parcel lockers are stored at the rural depot and do not contribute to the time-area requirements of the delivery scenario. It is noted that the parcel lockers were also occupying space in rural areas, which should normally be considered [13]. However, realistically speaking, land area usage in rural areas is generally less of a concern to delivery companies, city planning and politicians compared with area usage in densely populated cities. If the space requirement of modular lockers at the rural depot is considered, modular lockers would only have a slightly larger time-area requirement than fixed lockers, given that the time-area requirement of the locker adjustment trip is small per parcel (i.e., as low as 1 m^2^ × min per parcel).

**Figure 9:**
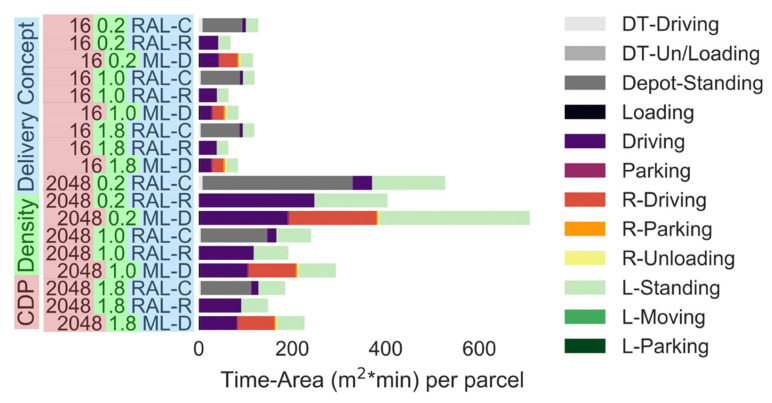
Time-area requirements of RAL and ML-D (number of CDPs: 16 or 2048). Parcel demand level: 0.2: 20% of the current demand, 1.0: current demand, 1.8: 80% increase in the demand.

Figure 9 shows the time-area requirements of the RAL and ML-D scenarios. Instead of changing the modular parcel lockers, in the ML-D simulation the required locker units were filled with a parcel at the rural depot and delivered to each CDP daily. Adopting the daily ML-D approach requires slightly less than double the time-area requirements than adjusting the lockers weekly (Figure 8 and Figure 9). The driving part of the delivery and pick up trip of the locker units accounts for the largest share of the time-area (i.e., 206 m^2^ × min for ML-D (Driving + R-Driving)), as much more space is required to transport parcels in locker units than the parcels themselves. By leaving the computer of each CDP in position overnight, the time-area requirement for the standing locker is increased by between 0.6% (1 CDP) and 51% (2048 CDP). ML-D requires always 36% (1 CDP) to 53% more time-area than RAL-R. If the RALs are stored at the city depot (i.e., RAL-C), ML-D requires 26% less time-area with one CDP or 22% more time-area with 2048 CDPs.

**Figure 10:**
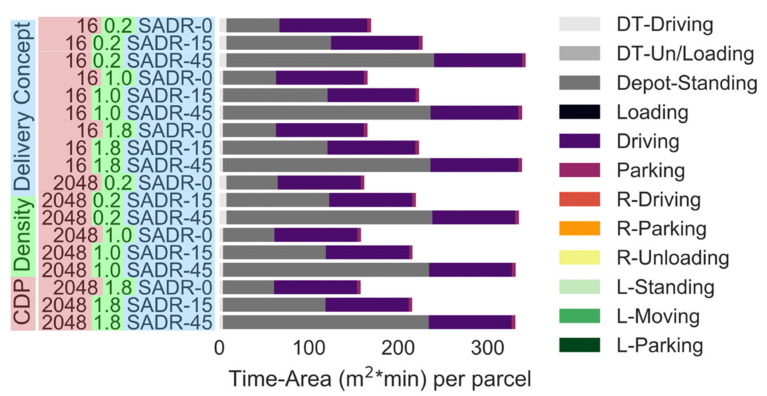
Time-area requirements of SADRs (number of CDPs: 16 or 2048). Parcel demand level: 0.2: 20% of the current demand, 1.0: current demand, 1.8: 80% increase in the demand. *x*-axis, different scale.

Parking at the city depot (i.e., 57 m^2^ × min–230 m^2^ × min depending on the average wait time) and driving (i.e., 93 m^2^ × min) are the two main contributors to the time-area requirements of SADRs (Figure 10). Unless the demand for SADRs is completely uniform throughout the day (i.e., SADRs are constantly delivering parcels during operating hours), the parking at the depot is the largest contributor to the time-area requirement. This is amplified by the fact that SADRs also need parking spots at the depot, even if they are not used during the low-demand season. The time-area requirements while driving are larger by factors ranging from 2.68 (CDP 256, density 0.2) to 142 (CDP 1 density 2.0) for SADRs when compared with a 7.5 t truck, as parcels can be stacked within the truck, and the average speed of delivery vans (i.e., driving speed) is faster compared to a SADR (i.e., walking speed).

### 4.3. Time-Area Requirements: Delivery Vehicles and Customers

**Figure 11:**
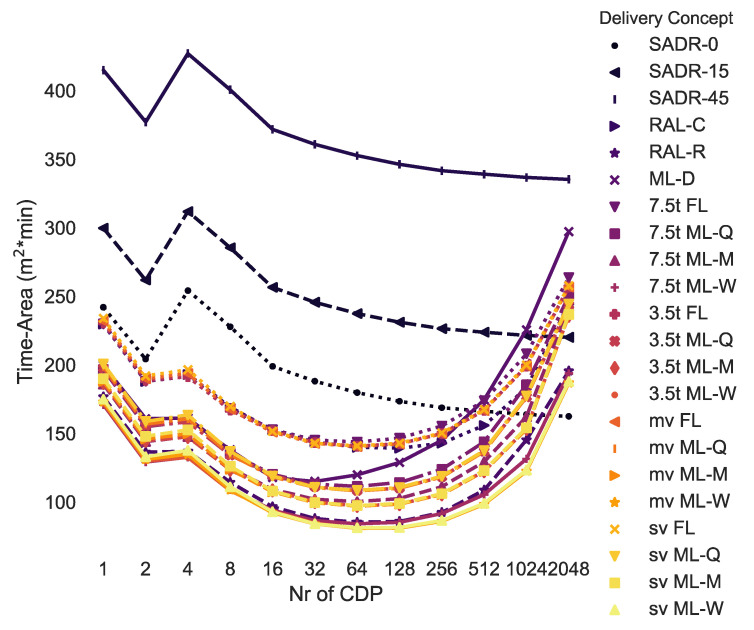
Time-area requirements for all delivery concepts depending on the number of CDPs (density: 1.0, customer: pedestrian).

Figure 11 compares the time-area requirements for each delivery concept depending on the number of CDPs. For example, delivering parcels by a medium van to 64 modular lockers that are adjusted weekly (i.e., 64 mv ML-W) is the best option if all customers walk to pick up their parcels, and the parcel demand level is 1.0 (see annotation on Figure 11). It is noted that using a small van, 3.5 t or 7.5 t van instead of a medium van with weekly adjusted modular lockers or a RAL-R performs similarly: within +0% to 7%.

Most of the curves in Figure 11 are non-monotonic, given that when the number of CDPs is low, the customer has to travel more and the delivery vehicle travels less. When many CDPs are used, the delivery vehicle travels more and the customer travels less. Hence, there is usually a minimum in the middle for tour-based deliveries. The curves of direct delivery services (i.e., SADRs) are overall monotonically decreasing.

Table 2 shows the best combination of CDPs and delivery concepts derived from the simulations. Given that the demand for parcels could vary in the future, the simulation has been conducted for 10 different demand levels and not just the three previously simulated demand levels. All concepts except for SADR-0 have been included, as it appeared unlikely that the delivery demand would be so uniform that SADRs would be employed continuously. As expected, when the parcel demand level is low (i.e., 0.2: 20% of the current demand), RAL-R is the best option (i.e., 32 CDPs: 74 m^2^ × min, 64 CDP: 87 m^2^ × min, 128 CDP: 109 m^2^ × min). This is due to the inhomogeneity of the parcel demand, and therefore, the low utilisation of parcel lockers. The higher the parcel demand level, the higher the utilisation of the delivery system, and modular parcel lockers then become the better option for a parcel demand level of 0.4 (i.e., 40% of the current demand) or larger. The tour scheduling algorithm developed in this research (see Section 3.3) always chooses the most suitable delivery vehicle up to a set maximum size. In most cases, the maximum van size should be medium or 3.5 t. The optimal number of CDPs is lowest for pedestrians (i.e., 32–128) and highest for cars (i.e., 128–1024) due to the time-area requirements of customers driving a car being around seven times higher compared to customers walking to pick up a parcel.

### 4.4. Sensitivity Analysis

A sensitivity analysis to determine the dependency of the result on the input variables was conducted for 12 factors. Table 3 shows the factors that have been varied in the sensitivity analysis.

**Figure 12:**
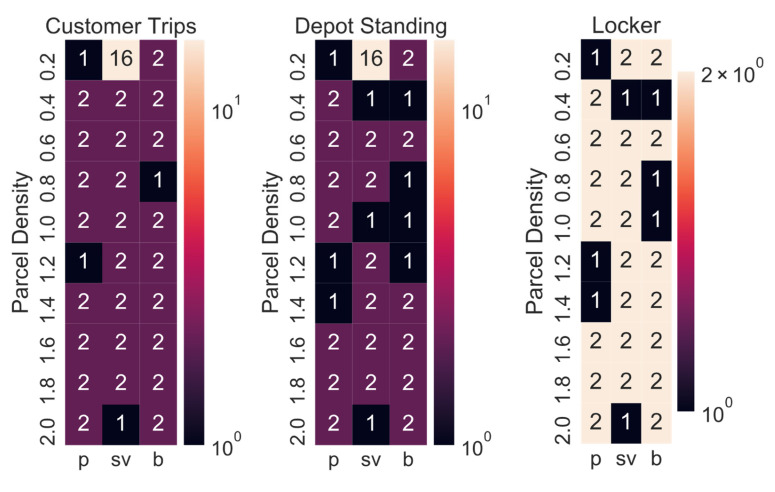
Increase in the optimal number of CDPs (2: doubled number of CDPs).

The optimal number of CDPs always stayed the same during the sensitivity analysis, apart from when the time-area requirements of the customer’s trip, or the depot standing, or the locker, was varied. Figure 12 shows the increase in the optimal number of CDPs. In most cases, the optimal number of CDPs is doubled if one of the three previously mentioned time-area requirements is doubled (i.e., customer trips), divided by ten (i.e., depot standing) or halved (i.e., lockers), respectively.

**Figure 13:**
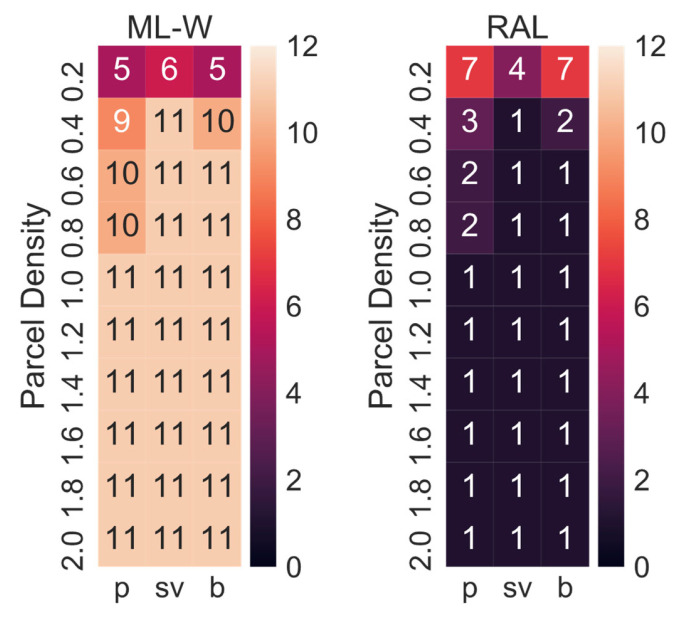
Numbers of times RAL and ML-W were the best options in the sensitivity analysis.

Figure 13 shows how often ML-W and RAL were the best options for each combination of customer mode choice and parcel demand level for the 12 variations in the sensitivity analysis. Unless the parking at the depot was varied in the sensitivity analysis, modular lockers adjusted weekly were always the best option for the current parcel demand level or higher demand levels. When the parcel demand level is very low, RALs are sometimes the better option. SADRs were only the best option in one case: the city depot is a multi-story building, the parcel demand level 0.2 and the customer picks up the parcel by car.

**Figure 14:**
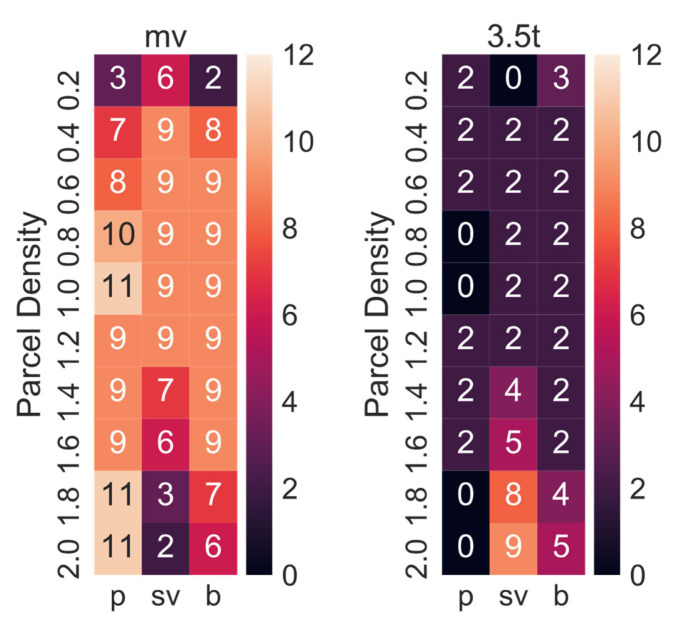
Numbers of times each maximum delivery van size (i.e., mv, 3.5 t) was the best option.

The best maximum delivery van size varied in the sensitivity analysis between medium vans (mv) and 3.5 t vans (Figure 14). The numbers do not add up to 12, as sometimes the best delivery concept did not include a delivery vehicle (i.e., RAL and SADR).

Overall, the sensitivity analysis shows that the simulation was relatively robust towards variations in 12 of the key parameters, especially when the parcel demand was the current level or higher.

## 5. Discussion

We simulated and compared the time-area requirements of various autonomous delivery concepts using real delivery demand data for one year. The study highlights the large time-area requirements if customers use cars to pick up parcels from lockers. The time-area requirement of a customer cycling to pick up a parcel is around three times as large as the time-area requirement of one who walks, and around half that of one who drives. When comparing the total time-area requirements (i.e., customer and delivery vehicle), delivery to 64 weekly-adjusted modular lockers using a medium van (i.e., 64 mv ML-W) is the best option if all customers walk to pick up their parcels. Using only small vans, up to 3.5 t or up to 7.5 t trucks instead to deliver to modular lockers, or a RAL-R, only increases the time-area requirements by up to 7%. Regardless of the demand for parcel deliveries being reduced or increased, delivery to weekly-adjusted modular lockers using a medium van (i.e., 64 mv ML-W) is in most cases still the best option. RAL-R is only the best option when a minimum number of parcels is delivered. This stayed relatively constant in the sensitivity analysis and only changed when the customer trips were changed, or either the depot or the CDPs locations were multi-story buildings. The optimal number of CDPs is mainly only affected by the mode of transport the customer chooses or the demand for parcel deliveries.

The results clearly highlight that modular lockers are an option that are worthwhile investigating from a time-area viewpoint. However, from a financial viewpoint, modular lockers might not be the best option [32].

The simulation also emphasised the importance of discouraging car usage to pick up parcels from a locker. To achieve this, a relatively dense network of CDPs needs to be built, given that asking customers to walk a 4.7 km roundtrip to pick up a parcel from a single CDP within a network might be unreasonable.

Even though SADRs performed well in other studies, for example, [4], SADRs did not perform well in this study. The main reason is that SADRs could only deliver one parcel at a time, in this study, making them a perfect choice for on-demand deliveries. However, SADRs struggle to compete against delivery vans, which can deliver a few hundred parcels at a time to parcel lockers. However, further studies are required to determine whether SADRs perform better for home delivery or whether having slightly larger SADRs, which can deliver two or maybe five parcels at a time, might improve the time-area requirements.

## 6. Conclusions

The paper proposes a decision support method to find the optimal combination in terms of the number of CDPs and delivery concept for autonomous parcel delivery based on the required time-area. Reducing the time-area of parcel delivery increases the effectiveness of space usage in cities, which reduces the space requirements for streets and parking and gives more space to housing and parks. This is a key factor to improving the quality of life in cities. The decision support method is a key tool for policymakers to steer the research into ADRs in the best direction, and later to incentivise the best autonomous delivery concept for their cities and discourage the harmful ones.

The decision support tool was applied to a dataset of all parcel delivery tours by a delivery company in London over one year (2-million parcels). Using a real-world dataset, instead of probabilistic scenarios, allows a more accurate simulation of daily or seasonal variations in the parcel deliveries. This paper compared fixed lockers, modular lockers, RALs and SADRs.

Modular lockers that are adjusted weekly are the best option for the current parcel demand level or for larger parcel demand levels, regardless of the customer modes. The same is the case even in the sensitivity analysis unless multiple lockers/RALs/SADRs can be stacked above each other at a depot. In that case, RAL-C is the best option. RAL-R is only the best option at the lowest parcel demand level. When parcels are delivered to modular lockers, the maximum van size should either be medium, or rarely, at high densities, 3.5 t. The optimal number of CDPs is smallest when the customer walks and at low densities, and is largest when the customer drives and at high densities.

## Figures and Tables

**Table 1:** Simulation specifications (source: adapted from [13]).

Mode	Speed Profile	$l_{i}\;(m)$	$s_{i}\;(m)$	$w_{i}\;(m)$	Following Rule	Max Parcels
Small van (sv)	Car	4.4	1	2.75	2	330
Medium van (mv)	Car	4.9	1	2.75	2	560
3.5 t van (3.5 t)	Car	5.9	1	2.75	2	960
7.5 t truck (7.5 t)	Car	6.7	3	2.75	6	3167
SADR	Pedestrian	0.678	0.197	0.875	1	1
RAL	Car	0.45	0	0.45	1	5, 15,…
Locker	-	0.45	0	0.45	-	5, 15,…
Bicycle (b)	Bicycle	1.8	0	1.5	2	1 customer
Pedestrian (p)	Pedestrian	0.875	0	0.875	1	1 customer
Small vehicle (sv)	Car	4.4	1	2.75	2	1 customer

**Table 2:** The best combination of the number of CDPs and delivery concepts for each density for each customer’s mode choice.

Density/Parcel Demand	Pedestrian (p)	Small Passenger Vehicle (sv)	Bicycle (b)
0.2	32 RAL-R	128 RAL-R	64 RAL-R
0.4	32 mv ML-W	256 mv ML-W	128 mv ML-W
0.6	64 mv ML-W	256 mv ML-W	128 mv ML-W
0.8	64 mv ML-W	256 mv ML-W	256 mv ML-W
1.0	64 mv ML-W	512 mv ML-W	256 mv ML-W
1.2	128 mv ML-W	512 mv ML-W	256 mv ML-W
1.4	128 mv ML-W	512 mv ML-W	256 mv ML-W
1.6	128 mv ML-W	512 mv ML-W	256 mv ML-W
1.8	128 mv ML-W	512 3.5 t ML-W	256 mv ML-W
2.0	128 mv ML-W	1024 3.5 t ML-W	256 mv ML-W

**Table 3:** Factors varied in the sensitivity analysis.

Sign	Factor	Increase
$l_{i}$	length of vehicle	+30%
$w_{i}$	width of the lane/right-of-way	+30%
$s_{i}$	safety distance between standing vehicles	doubling
$t_{s}$	following rule	doubling
$d_{i}$	distance due to detours	doubling
$t_{i}$	increase in driving duration due to traffic	doubled
$t_{i}$	increase in speed	halved
	loading time	doubled
	time-area requirement of the depot trips	doubled
	time-area requirement of the customer trips	doubled
	time-area requirement of the depot standing (e.g., using a multi-storey depot to reduce the required ground area)	1/10
	time-area requirement of lockers (e.g., lockers are located in two-storey buildings to halve the required ground area)	halved

## Data Availability

Map data are copyrighted by OpenStreetMap contributors and available from https://www.openstreetmap.org (accessed on 3 August 2021). Parcel delivery data provided under the Open Government Licence v3.0 maintained by Greater London Authority online, available at: https://data.london.gov.uk/dataset/key-performance-indicators-of-demonstrator-freight-delivery-performance-with-electric-vans-in-central-london (accessed on 3 February 2020).
